# Associations of dietary patterns with blood pressure and markers of subclinical arterial damage in adults with risk factors for CVD

**DOI:** 10.1017/S1368980021003499

**Published:** 2021-08-16

**Authors:** Vicky Tzelefa, Christiana Tsirimiagkou, Antonios Argyris, George Moschonis, George Perogiannakis, Maria Yannakoulia, Petros Sfikakis, Athanase D Protogerou, Kalliopi Karatzi

**Affiliations:** 1 Department of Nutrition and Dietetics, School of Health Science and Education, Harokopio University of Athens, Greece; 2 Cardiovascular Prevention & Research Unit, Clinic & Laboratory of Pathophysiology, Department of Medicine, National and Kapodistrian University of Athens, Greece; 3 Department of Dietetics, Nutrition and Sport, School of Allied Health, Human Services and Sport, La Trobe University, Bundoora, VIC, Australia; 4 Cardiovascular Research Laboratory, 1st Department of Propaedeutic and Internal Medicine, Athens University Medical School, Laiko Hospital, Athens, Greece; 5 Hellenic Foundation for Cardiovascular Health and Nutrition, Athens, Greece; 6 Department of Food Science and Human Nutrition, Agricultural University of Athens, Greece Iera Odos 75, 118 55 Athens, Greece

**Keywords:** Dietary patterns, Blood pressure, Arterial stiffness, Pressure wave reflection, CVD

## Abstract

**Objective:**

Unhealthy diet is a modifiable risk factor leading to subclinical arterial damage (SAD), high BP and CVD. It was aimed to investigate the possible associations of dietary patterns (DPs) with SAD in adults having multiple CVD risk factors.

**Design:**

Dietary intake was evaluated through two 24-h dietary recalls and principal component analysis was used to identify DPs. Oscillometry, applanation tonometry with pulse wave analysis and carotid ultrasound were used to assess peripheral and aortic BP, arterial stiffness and pressure wave reflections.

**Setting:**

Laiko University Hospital, Athens, Greece.

**Participants:**

A total of 470 individuals (53·1 ± 14·2 years) with CVD risk factors were enrolled.

**Results:**

A pattern characterised by increased consumption of whole-grain cereals, white meat and reduced consumption of sugar was positively associated with common carotid compliance (*β* = 0·01, 95 % CI 0·00, 0·01), whereas a pattern high in refined cereals, red and processed meat was positively associated with brachial but not aortic systolic pressure (*β* = 1·76, 95 % CI 0·11, 3·42) and mean arterial pressure (MAP) (*β* = 1·18, 95 % CI 0·02, −2·38). Low consumption of low-fat dairy products, high consumption of full-fat cheese and butter was positively associated with MAP (*β* = 0·97, 95 % CI 0·01, 1·95). Increased consumption of vegetables, fruits, fresh juices, fish and seafood was inversely associated with augmentation index (AIx) (*β* = -1·01, 95 % CI -1·93, −0·09).

**Conclusion:**

Consumption of whole grains, white meat, fruits/vegetables, fish/seafood and avoidance of sugar was associated with improved SAD. Preference in refined grains, red/processed meat, high-fat cheese/butter and low intake of low-fat dairy products were associated with BP elevation. Future studies are needed to confirm the present findings.

CVD is currently the leading cause of death globally, which is projected to cause about 23·6 million deaths by 2030^(1)^. Subclinical arterial damage (SAD) precedes the onset of CVD for many decades. Some of the main pathophysiological steps in the development of CVD include SAD associated with premature arterial stiffening and alteration in peripheral and central haemodynamics, such as in brachial and aortic blood pressure (BP) and pressure wave reflections^(2–4)^. Early assessment of SAD would facilitate CVD risk stratification and would also lead to improved primary CVD prevention^(5)^.

Numerous non-invasive vascular biomarkers, reﬂecting different types of arterial damage, are commonly used in clinical practice. As regards to macrocirculation, stiffness of elastic arteries such as the aorta and the carotid arteries predicts CVD risk^(6)^ and improves risk stratification^(7)^. The carotid-to-femoral (cf) pulse wave velocity (PWV), estimated by applanation tonometry and pulse wave analysis, is considered as the gold-standard biomarker for arterial stiffness assessment in clinical practice^(6)^. Moreover, assessment of carotid elasticity (distensibility and compliance) is of added clinical value too^(8,9)^. Pressure wave reflections, as assessed by augmentation index (AIx), have been shown to provide clinical and pathophysiological information on CVD^(10,11)^. Finally, non-invasive assessment of aortic BP and pulse pressure amplification (PPamp) has been repeatedly shown to provide further predictive information beyond the classical brachial BP^(10–12)^.

One of the most important modifiable risk factors of CVD is unhealthy diet. During the past years, studies were investigating the separate effects of single nutrients, food items or food groups on the risk of chronic disease development^(13)^. However, human diet does not include isolated food items, but a combination of them, providing various nutrients that may interact in a non-predictable, even synergistic way^(13,14)^. Therefore, studying dietary patterns (DPs) instead of specific food items or nutrients has emerged as an alternative way to investigate the effect of diets as clusters that combine the consumption of specific foods on chronic diseases risk, as this approach enables a more pragmatic evaluation of everyday diet.

There are several studies investigating the associations between *a priori* DPs, such as the Mediterranean diet, DASH diet, Western diet and markers of subclinical vascular dysfunction^(15–17)^. However, *a priori* DPs are based on existing knowledge about the relationships between food, nutrients and disease and this analysis evaluates adherence to a specific pattern or recommendation. *A posteriori* DPs are based on statistical methods and can incorporate actual characteristics of individuals’ choices in relation to the development of a disease^(18)^ and they provide new insight on the relationship between diet and disease. Limited data are available on the association between *a posteriori* defined DPs and the early dysfunction of the microcirculation which is a common pathophysiological mechanism in the development of insulin resistance, CVD and type 2 diabetes mellitus^(19,20)^.

In a previous study of our team, a DP characterised by increased consumption of vegetable oils, poultry fish and seafood was positively associated with both functional and anatomic capillary density and a second DP with increased consumption of sweets was inversely associated with functional and anatomic capillary density^(19)^. Even more limited data on the association between *a posteriori* defined DPs and SAD on the macrocirculation exist. A prospective cohort study found a significantly positive association between PWV and a DP characterised by increased meat and poultry, processed meat and alcohol consumption and low fibre intake^(21)^ and, also, a recent cross-sectional study indicated that a DP characterised by high consumption of vegetables, seafood, seaweed, fruit and pulse is inversely associated with arterial stiffness measured by PWV^(22)^.

Given the existence of limited and rather inconsistent data on the association of DPs with early markers of SAD on the macrocirculation, the aim of the present study was to investigate the potential associations between DPs and vascular biomarkers of macrocirculation in adults free of CVD, with risk factors for CVD.

## Materials and methods

### Study design and population

The current study is part of a large cross-sectional research conducted at the Cardiovascular Research Laboratory of the First Department of Propaedeutic and Internal Medicine, Laiko University Hospital, Athens, Greece. The study population consisted of 470 subjects having one or more CVD risk factors (obesity, hypertension, smoking, dyslpidemia, diabetes). Participants’ characteristics are presented in Table 1. Participants with established CVD (pre-existing coronary artery disease, stroke or peripheral arterial disease) were excluded from the analysis. All participants visited the laboratory early in the morning, having refrained from food and any vasoactive medication and after 30 min of acclimatisation in a quiet, temperature-controlled environment (22–25°C), they were subjected to anthropometric and vascular assessment. All participants provided written informed consent according to the World Health Organization statement on ethical principles for medical research involving human subjects developed in Helsinki and the protocol was approved by the ‘Laiko’ Hospital’s institutional review board.

**Table 1:** Descriptives of study population

	Mean	sd
Population		
* n*	470	–
Men		
* n*	235	
* *%	50·0	
Age, years	53·1	14·2
BMI, kg/m^2^	28·4	5·5
Smoking status		
* *Never smoker		
* *%	46·8	–
* *Former smoker		
* *%	31·5	–
* *Current smoker		
* *%	21·7	–
Obesity		
* *%	30·6	
Diabetes		
* *%	31·9	–
Hypertension		
* *%	53·2	–
Dyslipidemia		
* *%	44·9	–
Brachial systolic BP, mmHg	127·8	16·0
Brachial diastolic BP, mmHg	77·1	9·3
Aortic systolic BP, mmHg	117·4	17·5
MAP, mmHg	89·0	11·2
PPamp	1·3	0·2
Heart rate, bpm	68·3	11·0
Cf-PWV, m/s	8·5	2·1
AIx @75		
* *%	25·1	12·5
Common carotid compliance, mm^2^kPa	0·12	0·06
Common carotid distensibility, kPa^−1^	0·004	0·0023

BP, blood pressure; MAP, mean arterial pressure; PP, pulse pressure; PPamp, pulse pressure amplification; Cf-PWV, carotid-femoral pulse wave velocity; Aix, augmentation index.

Values are presented as mean or frequencies (%) and SD.

### Definition of CVD risk factors

Hypertension was defined as the use of antihypertensive drugs and/or office BP measurement higher than 139/89 mmHg (average of three sequential readings). BMI was calculated as weight/(height)^2^ (kg/m^2^) and obesity was considered as BMI > 30 kg/m^2^. Current smoking was defined using at least one cigarette/d each day of the week; ex-smoking was defined as discontinuation of smoking for >6 months. Dyslipidemia was defined by treatment with lipid-modifying drugs or LDL cholesterol level >160 mg/dl. Diabetes was defined as glucose 126 mg/dl or Hb A1c > 6·5 % and/or glucose-lowering treatment.

### Blood pressure measurement

Office brachial BP measurements were performed with a validated automated oscillometric device (Microlife WatchBP Office, Microlife AG, Widnau, Switzerland). Each subject was seated for 10 min in a supine position and then three consecutive measurements were recorded with 1-min interval between them. The average of BP values was used in statistical analysis. Office aortic BP was assessed through applanation tonometry of the radial artery and the use of validated transfer function (Sphygmocor, AtCor Medical, Sydney, Australia). Pulse pressure (PP) is the difference between the systolic and diastolic, and was calculated; 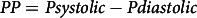 . PP is necessary for the calculation of mean arterial pressure (MAP). MAP was estimated by the formula


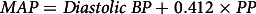
 ^(23)^. PPamp was assessed as the ratio of brachial/aortic PP, as previously suggested^(24)^.

### Estimation of arterial stiffness, compliance and distensibility

Cf-PWV was measured according to a standard methodology described elsewhere using the SphygmoCor apparatus (AtCor Medical, Sydney, Australia)^(7,25)^. Briefly, cf-PWV is calculated by the ratio of the estimated pulse transit time and the distance travelled by the pressure wave between the two recording sites. The same operator performed all measurements and each subject was in a supine position. Pressure waves were first recorded at the carotid artery and then, within a few seconds, at the femoral artery. The time delay between the two waves (transit time) was determined using registration with a simultaneously recorded ECG. At least two repeated measurements of cf-PWV were performed, and their average value was used in the analysis in accordance with previous recommendations^(26)^. Based on Bramwell and Hill theory, arterial compliance was estimated based on PWV:


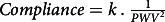
 where k is a coefficient that accounts for the contribution of local geometry and wave speed of each segment of the arterial tree^(27)^. Finally, common carotid artery distensibility was calculated according to the following formula: *Distensibility* = (2Δ*D* × *D* + Δ *D* ^2^)/(*PP* × *D* ^2^) × (10^− 3^/kPa), where D is the arterial diameter, ΔD is the distension (change in arterial diameter during heart cycle)^(28)^.

### Estimation of pressure wave reflections

The AIx was also assessed, as an index of pressure wave reflections, as previously described^(29)^ and normalised for the heart rate of 75 bpm (AI@75) due to the strong dependence of this index on heart rate.

### Dietary assessment

Two 24-h dietary recalls were conducted via telephone interview by a trained dietitian. The time interval between the two recalls was at least 1 week. The study participants were asked to report, as detailed as possible, the type and number of foods and beverages consumed during the previous day. This procedure was performed for 1 weekday and 1 weekend day (nonconsecutive days). At first, data from the 24-h recalls were grouped into food groups (forty food groups derived from the dietary intake assessment). Subsequently, dietary intake was analysed in terms of energy, macro- and micronutrients content using Nutritionist pro Software (Axxya Systems Nutritionist Pro TM 2011), which was expanded by adding traditional Greek foods and Greek recipes^(30)^.

### Statistical analysis

Statistical analysis was performed using the SPSS statistical package (IBM, version 21.0; IBM). Normality of the variables was tested using the Kolmogorov–Smirnov test. Continuous variables are presented as mean ± SD (SD) and categorical variables as frequencies. The level of statistical significance was set at *P* < 0·05. All tests were two-tailed.

Principal components analysis (PCA) with all forty food groups derived from the dietary assessment was used to identify DPs using the *a posteriori* approach. The Kaiser–Meyer–Olkin (KMO) criterion was applied, and it was equal to 0·55. The orthogonal rotation (varimax option) was used to derive optimal non-correlated components (DPs) and the Bartlett’s method was used to estimate factor scores^(31,32)^. The selection of the optimal number of components was based on an eigen value>1 (Kaizer criterion) and it was further corroborated by visual assessment of the scree plot, retaining only components on the steep slope. If one of the initial food groups correlated with more than one component, it participated in the interpretation of the dietary pattern in which it displayed the highest coefficient value.

Multiple linear regression analysis was performed to determine the independent associations of the five DPs identified with vascular dysfunction markers. The major classical CVD risk factors (age, sex, smoking, dyslipidemia, hypertension^(33)^) were used as potential confounders in multivariable regression models, as follows: model 1: unadjusted; model 2: adjusted for age and sex; model 3: adjusted for age, sex, smoking, diabetes, dyslipidemia, energy intake, drugs for hypertension, diabetes and dyslipidemia. Also, we ran model 3 either adding BMI as a confounder or substituting total energy intake with BMI. The results are presented as unstandardised b coefficients (*β*) and confidence intervals (CI).

## Results

A total of 2090 subjects with cardiovascular risk factors were included in the initial phase of the study, of which 1031 had full data regarding dietary assessment. After exclusion of people with established CVD or autoimmune diseases, a sample of 470 CVD-free subjects with one or more CVD risk factors was included in the analysis. Descriptive and clinical characteristics of the study population are reported in Tables 1 and 2.

**Table 2:** The mean daily consumption of the studied food groups in servings/d in our population

	Mean	sd
Energy intake, (kJ/d)	7228·5	2605·9
Carbohydrates (g/d)	177·9	72·3
Proteins (g/d)	71·8	32·0
Fat (g/d)	78·0	35·3
Carbohydrates (% of daily energy intake)	41·6	10·1
Proteins (% of daily energy intake)	16·9	5·1
Fat (% of daily energy intake)	40·4	9·3
Saturated fat (g/d)	22·5	11·1
Monounsaturated fat (g/d)	36·1	18·9
Polyunsaturated fat (g/d)	11·2	6·9
Sodium (g/d)	1735·6	957·2
Potassium (g/d)	2354·9	977·8
Whole-grain products* (1/2 cup or otherwise stated)	1·1	1·4
Refined grain products† (1/2 cup or otherwise stated)	3·0	2·3
White meat‡, (60 g/d)	0·5	0·7
Red meat§, (60 g/d)	1·0	1·0
Processed meat‖, (30 g/d)	0·3	0·6
Fish, (60 g/d)	0·4	0·9
Sea food, (60 g/d)	0·1	0·3
Low-fat dairy product¶, (glass/d)	0·4	0·5
Full-fat cheese**, (30 g/d)	1·0	1·1
Butter, (1 tsp/d)	0·1	0·5
Vegetables (1/2 cup/d)	2·5	1·9
Fruits and fresh juices (1/2 cup/d)	1·5	1·6
Sugar, (1 tsp/d)	0·7	1·1
Non-diet soft drinks (glass/d)	0·1	0·2
Heavy alcoholic drinks††, (30 ml/d)	0·2	0·8

* Whole wheat bread (one slice), Greek sesame bread ring (half), whole wheat flour, brown rice, whole-grain pasta, whole-grain cereals, oats, muesli, wheat bran.

† White bread (one slice), pita bread (half), white flour, white rice, pasta, corn flakes, white cereals.

‡ Chicken, turkey, duck, rabbit.

§ Beef, pork, lamb, goat, offal.

‖ Precooked or cured meat like ham, salami, sausages, bacon.

¶ Skimmed milk, light evaporated milk, soy milk, low-fat milk (0–2 %), low-fat yoghurt (0–2 %).

** Cheese with > 20 % fat like cream cheese, feta, mozzarella, gruyere, kefalograviera, kefalotyri, metsovone, parmesan, cheddar, edam.

†† Drinks with >40 % alcohol like Gin, vodka, whiskey, rum, brandy, liqueur, martini.

Values are presented as mean and SD.

Table 3 summarises the loadings of the factors retained from the PCA and indicated five DPs explaining 50 % of the total variance regarding the examined variables. These patterns were characterised as follows: increased consumption of whole-grain cereal products and white meat and reduced consumption of sugar (DP 1); increased consumption of refined cereal products, red meat and processed meat (DP 2); reduced consumption of low-fat dairy products and increased consumption of full-fat cheese and butter (DP 3); increased consumption of vegetables, fruits and fresh juices, fish and seafood (DP 4); increased consumption of heavy alcoholic drinks and non-diet soft drinks with sugar (DP 5).

**Table 3:** Factor loadings for the five dietary patterns derived from principal component analysis on food groups consumed in the study population

Predictors (food groups)	Component				
	1	2	3	4	5
Whole-grain products*	0·669				
White meat†	0·602				
Sugar	−0·451				
Refined grain product‡		0·742			
Red meat§ and processed meat‖		0·629			
Low-fat dairy products¶			−0·560		
Full-fat cheese**			0·606		
Butter			0·639		
Vegetables				0·653	
Fruits and fresh juices				0·627	
Fish and seafood				0·593	
Heavy alcoholic drinks††					0·795
Non-diet soft drinks					0·466
Explained variance	10·94	10·60	9·63	9·59	9·24

* Whole wheat bread, Greek sesame bread ring, whole wheat flour, brown rice, whole-grain pasta, whole-grain cereals, oats, muesli, wheat bran.

† Chicken, turkey, duck, rabbit.

‡ White bread, pita bread, white flour, white rice, pasta, corn flakes, white cereals.

§ Beef, pork, lamb, goat, offal.

‖ Precooked or cured meat like ham, salami, sausages, bacon.

¶ Skimmed milk, light evaporated milk, soy milk, low-fat milk (0–2 %), low-fat yoghurt (0–2 %).

** Cheese with > 20 % fat like cream cheese, feta, mozzarella, gruyere, kefalograviera, kefalotyri, metsovone, parmesan, cheddar, edam

†† Drinks with >40 % alcohol like Gin, vodka, whiskey, rum, brandy, liqueur, martini.

Variables with the highest factor loading (>|0·4|) within the component.

Table 4 presents the associations between the identified DPs and brachial systolic and diastolic BP, aortic systolic BP, MAP, PPamp. After adjustment for potential confounders, the following associations were observed. DP 2 was positively associated with brachial systolic BP (*β* = 1·76, 95 % CI 0·11, 3·42; model 3) and MAP (*β* = 1·18, 95 % CI 0·02, 2·38; model 3). DP 3 was positively associated with MAP (*β* = 0·97, 95 % CI 0·01, 1·95; model 3).

**Table 4:** Associations of the derived dietary patterns with arterial pressure indices

Components	Model 1*		Model 2†		Model 3‡	
	*β*	95 % CI	*β*	95 % CI	*β*	95 % CI
Brachial systolic pressure						
DP 1§	−0·22	−1·68, 1·24	0·09	−1·29, 1·47	0·04	−1·38, 1·39
DP 2‖	0·05	−1·41, 1·50	1·15	−0·35, 2·65	1·76	0·11, 3·42
DP 3¶	0·30	−1·16, 1·76	0·18	−1·20, 1·56	0·45	−0·91, 1·82
DP 4**	1·21	−0·25, 2·67	0·82	−0·56, 2·21	1·22	−0·21, 2·64
DP 5††	−0·49	−1·95, 0·96	−0·37	−1·78, 1·04	0·24	−1·17, 1·65
Aortic systolic pressure						
DP 1	−1·12	−2·71, 0·46	−0·58	−2·02, 0·86	−0·68	−2·15, 0·80
DP 2	−1·26	−2·85, 0·33	0·80	−0·76, 2·37	1·47	−0·30, 3·24
DP 3	0·56	−1·02, 2·15	0·57	−0·87, 2·02	0·82	−0·62, 2·27
DP 4	0·60	−0·99, 2·18	0·26	−1·20, 1·70	0·67	−0·84, 2·19
DP 5	−0·76	−2·34, 0·83	−0·21	−1·69, 1·27	0·28	−1·22, 1·78
Brachial diastolic pressure						
DP 1	−0·98	−1·81, −0·15	−1·02	−1·84, −0·21	−0·71	−1·55, 0·13
DP 2	0·85	0·02, 1·69	0·52	−0·37, 1·41	0·90	−0·10, 1·91
DP 3	0·67	−0·16, 1·50	0·46	−0·36, 1·28	0·69	−0·13, 1·52
DP 4	0·18	−0·66, 1·01	−0·10	−0·92, 0·72	0·20	−0·66, 1·07
DP 5	0·32	−0·52, 1·15	−0·04	−0·88, 0·79	0·26	−0·59, 1·11
Mean arterial pressure						
DP 1	−0·83	−1·85, 0·19	−0·68	−1·66, 0·30	−0·56	−1·56, 0·44
DP 2	0·23	−0·79, 1·25	0·70	−0·37, 1·76	1·18	0·02, 2·38
DP 3	0·87	−0·15, 1·89	0·74	−0·24, 1·72	0·97	0·01, 1·95
DP 4	0·46	−0·56, 1·48	0·17	−0·81, 1·16	0·48	−0·55, 1·51
DP 5	−0·33	−1·35, 0·68	−0·38	−1·39, 0·62	0·01	−1·01, 1·02
Pulse pressure amplification						
DP 1	0·76	−0·31, 1·83	1·04	0·02, 2·06	0·59	−0·42, 1·61
DP 2	−0·84	−1 91, 0·24	0·25	−0·86, 1·36	0·63	−0·59, 1·85
DP 3	0·03	−1·04, 1·10	0·05	−0·97, 1·07	0·10	−0·90, 1·10
DP 4	0·63	−0·44, 1·69	0·47	−0·55, 1·49	0·62	−0·42, 1·67
DP 5	−0·82	−1·89, 0·25	−0·51	−1·55, 0·53	−0·21	−1·24, 0·82

DP, Dietary pattern; *β*, unstandardised *β* coefficient; CI, confidence intervals.

* Model 1: including all dietary patterns.

† Model 2: model 1 and also adjusted for age and sex.

‡ Model 3: model 2 and also adjusted for smoking, diabetes, dyslipidemia, energy intake, drugs for hypertension, diabetes and dyslipidemia.

§ DP1: high consumption of whole-grain products and white meat and lower consumption of sugar.

‖ DP2: high consumption of refined grain products, red meat and processed meat.

¶ DP3: high consumption of full-fat cheese and butter and lower consumption of low-fat dairy products.

** DP4: high consumption of vegetables, fruits, fresh juices, fish and seafood.

†† DP5: high consumption of heavy alcoholic drinks and non-diet soft drinks.

Table 5 shows the associations of the derived DPs with AIx adjusted for 75 beats/min (AI@75). Only DP 4 was negatively associated with AIx after adjustment for all confounders (*β* = -1·01, 95 % CI-1·93, −0·09; model 3).

**Table 5:** Associations of the derived dietary patterns with pressure wave reflections (augmentation index adjusted for 75 beats/min (AI@75))

Components	Model 1*		Model 2†		Model 3‡	
	*β*	95 % CI	*β*	95 % CI	*β*	95 % CI
AI@75						
DP 1§	−1·16	−2·25, −0·07	−0·47	−1·34, 0·40	−0·52	−1·42, 0·38
DP 2‖	−3·26	−4·35, −2·16	−0·21	−1·16, 0·73	−0·10	−1·18, 0·97
DP 3¶	−0·12	−1·22, 0·97	0·35	−0·52, 1·22	0·35	−0·53, 1·23
DP 4**	−1·36	−2·46, −0·27	−1·14	−2·02, −0·27	−1·01	−1·93, −0·09
DP 5††	−0·62	−1·72, 0·47	0·79	−0·10, 1·68	0·66	−0·25, 1·57

DP, Dietary pattern; *β,* unstandardised *β* coefficient; CI, confidence intervals.

* Model 1: including all dietary patterns.

† Model 2: model 1 and also adjusted for age and sex.

‡ Model 3: model 2 and also adjusted for smoking, diabetes, dyslipidemia, hypertension, energy intake, drugs for hypertension, diabetes and dyslipidemia.

§ DP1: high consumption of whole-grain products and white meat and lower consumption of sugar.

‖ DP2: high consumption of refined grain products, red meat and processed meat.

¶ DP3: high consumption of full-fat cheese and butter and lower consumption of low-fat dairy products.

** DP4: high consumption of vegetables, fruits, fresh juices, fish and seafood.

†† DP5: high consumption of heavy alcoholic drinks and non-diet soft drinks.

Finally, Table 6 presents the associations between the identified DPs and common carotid compliance, distensibility and PWV. DP 1 was found to be positively associated with common carotid compliance (*β* = 0·01, 95 % CI 0·00, 0·01; model 3). No other associations were found between the remaining DPs and indices of arterial elasticity after adjustment for confounders.

**Table 6:** Associations of the derived dietary patterns with arterial elasticity of the right common carotid and the aorta

Components	Model 1*		Model 2†		Model 3‡	
	*β*	95 % CI	*β*	95 % CI	*β*	95 % CI
Common carotid compliance						
DP 1§	0·08	0·00, 0·01	0·01	0·00, 0·01	0·01	0·00, 0·01
DP 2‖	0·01	0·01, 0·02	0·00	0·00, 0·01	0·00	0·00, 0·01
DP 3¶	0·00	0·00, 0·01	0·00	−0·01, 0·01	0·00	−0·01, 0·01
DP 4**	0·00	0·00, 0·01	0·01	0·00, 0·01	0·01	0·00, 0·01
DP 5††	0·00	0·00, 0·01	0·00	0·00, 0·01	0·00	0·00, 0·01
Common carotid distensibility						
DP 1	0·00	0·00, 0·00	0·00	0·00, 0·00	0·00	0·00, 0·00
DP 2	0·00	0·00, 0·00	0·00	0·00, 0·00	0·00	0·00, 0·00
DP 3	0·00	0·00, 0·00	0·00	0·00, 0·00	0·00	0·00, 0·00
DP 4	0·00	0·00, 0·00	0·00	0·00, 0·00	0·00	0·00, 0·00
DP 5	0·00	0·00, 0·00	0·00	0·00, 0·00	0·00	0·00, 0·00
Pulse wave velocity						
DP 1	0·01	−0·19, 0·22	0·11	−0·05, 0·27	−0·03	−0·17, 0·12
DP 2	−0·19	−0·39, 0·01	0·05	−0·12, 0·22	0·08	−0·10, 0·25
DP 3	−0·11	−0·31, 0·01	−0·11	0·27, 0·05	−0·12	−0·26, 0·03
DP 4	0·11	0·09, 0·20	0·07	−0·14, 0·17	0·05	−0·10, 0·20
DP 5	−0·11	−0·31, 0·08	−0·41	−0·22, 0·09	−0·02	−0·16, 0·13

DP, Dietary pattern; *β,* unstandardised *β* coefficient; CI, confidence intervals.

* Model 1: including all dietary patterns.

† Model 2: model 1 and also adjusted for age and sex.

‡ Model 3: model 2 and also adjusted for smoking, diabetes, dyslipidemia, hypertension, energy intake, drugs for hypertension, diabetes and dyslipidemia.

§ DP 1: high consumption of whole-grain products and white meat and lower consumption of sugar.

‖ DP 2: high consumption of refined grain products, red meat and processed meat.

¶ DP 3: high consumption of full-fat cheese and butter and lower consumption of low-fat dairy products.

** DP 4: high consumption of vegetables, fruits, fresh juices, fish and seafood.

†† DP 5: high consumption of heavy alcoholic drinks and non-diet soft drinks.

No association between DP 5 and any of all the mentioned indices of subclinical vascular damage was identified.

Additional analyses were performed using model 3 by either adding BMI as a confounder or by substituting total energy intake with BMI. Both analyses revealed no alterations compared with the results already presented.

Finally, the size effect of the derived dietary patterns on systolic blood pressure, mean blood pressure (for pattern 2), mean blood pressure (for pattern 3), augmentation index (for pattern 4) and common carotid compliance (for pattern 1) when quantified relatively to the effect of age, were 32 %, 77·7 %, 88·8 %, 66·7 % of the partial *R* ^2^ of age, respectively.

## Discussion

In the present study, we investigated the associations of *a posteriori* DPs with early markers of SAD of the macrocirculation in adults free of CVD but with multiple CVD risk factors. We applied methodologies that extensively assessed elasticity at various arterial beds, pressure wave reflections and BP at the peripheral and central arteries. According to the analysis, five major DPs were identified. Two of them, (DP 1 and DP 4) had favourable and two (DP 2 and DP3) had unfavourable associations with biomarkers of SAD, while one showed no association with any biomarker.

**DP 1** (i.e., increased consumption of whole-grain products, white meat and reduced consumption of sugar) was marginally positively associated with common carotid compliance. Regarding increased intake of whole-grain products, it is already known that high fibre intake is associated with lower CVD risk^(34,35)^. Also, a longitudinal cohort study amongst 373 participants followed for 24 years, showed that subjects with less stiff carotid arteries consumed more fibre during the 24-year study than those with stiffer carotid arteries, as defined by distensibility and compliance measurements^(36)^. As regards to sugar intake, it is evident that increased consumption of sugar is positively associated with insulin resistance and consequently with arterial stiffness^(37)^, as the fast rate of absorption that follows the consumption of sugar may unfavourably influence blood glucose control, which may lead to hyperinsulinemia and peripheral insulin resistance^(38)^. Of note in the present study, we found a marginal association of this DP only with local carotid elasticity but not with segmental aortic stiffness. We can only speculate on the aetiology of this finding- since there are no other data in the literature that as previously extensively discussed may relate to the differences observed between arterial elastic properties from arterial site to site^(39)^. Also, it should be noted that the association found between DP1 and common carotid compliance is relatively small, yet previous studies have shown that per 1 sd change in common carotid compliance (i.e., 0·07) is associated with even 50 % higher all-cause mortality. Therefore, a change by 0·01 is of potential clinical importance^(40)^.

**DP 2** (increased consumption of refined grain products, red meat and processed meat) was found to be positively associated with brachial systolic BP and MAP. Previous findings on the association of refined-grain intake with BP have been highly inconsistent, because there are studies reporting association^(41)^ and others reporting no significant association^(42)^. A recent meta-analysis was conducted investigating the associations of 12 food groups (including grains, fruits and vegetables, nuts, legumes, eggs, dairy products, fish, red meat and beverages) with the risk of hypertension^(43)^. There was an inverse association between consumption of 30 g whole grains/d and the risk of hypertension and a relevant positive association between, red and processed meat daily consumption and the risk of hypertension. However, there were no significant associations regarding refined grains daily consumption^(43)^. Also, high consumption of red, white and processed meat was directly associated with systolic BP in the Nurses’ Health Study, which is partly explained by the fact that processed meat is high in sodium^(41)^. The mechanisms through which the intake of red meat may adversely impact on BP remain unclear and likely reflect competitive effects on BP amongst multiple dietary components. Saturated fat, cholesterol, animal protein and heme iron are the major nutrients of red meat. Direct relations of saturated fat and cholesterol with BP have been repeatedly reported in large cohort studies^(44,45)^.

**DP 3** (reduced consumption of low-fat dairy products and increased consumption of full-fat cheese and butter) was positively associated with MAP. Previous studies have shown that consumption of a diet rich in dairy products (either high or low fat) is associated with a reduction in BP^(46–49)^ and they are in agreement with the present findings. On the other hand, a recent meta-analysis indicated that a daily increase in dairy products intake by 200 g is negatively associated with the risk of hypertension, and there were no significant differences between low and high-fat dairy products^(43)^. Concerning full-fat cheese and butter it is known that are high in sodium and saturated fat and there are previous epidemiologic studies, that have shown a positive association between BP, sodium and saturated fats intake^(44,45,50)^. Also, there are cross-sectional studies showing positive associations between cheese and BP^(51,52)^. On the contrary, a randomised, cross-over study explored the effects of three different diets on BP: an intervention arm with a high-fat cheese diet and two control arms with either non-dairy products, high-fat meat diet or a non-dairy products, low-fat, high-carbohydrate diet. The results had shown no significant differences between the three diets^(53)^, which is not in line with the relevant findings in the present study. Also, recent findings indicate that there is an inverse association of cheese and other dairy products with CVD risk, which makes us sceptical regarding its association with BP^(54)^. There aren’t many studies investigating the association between butter and BP. In a recent randomised clinical trial conducted in 160 individuals three intervention arms were used, one with extra virgin coconut oil, one with extra virgin olive oil and one with unsalted butter. Participants were asked to consume 50 g daily of one of the three mentioned fats for 4 weeks without making other changes to their usual diet with no significant differences regarding changes in systolic or diastolic BP between the three intervention groups^(55)^. Another observational study (MONICA), concluded that there is no significant association with either SBP or DBP of dairy products consumption (with or without butter)^(56)^. These findings are in contrast with the present study.

**DP 4** (increased consumption of vegetables, fruits and fresh juices, fish and seafood) was inversely associated with AIx. A recent meta-analysis showed that there are undefined pathogenic mechanisms connected with vascular damage and insulin resistance. Yet, it is evident that vascular dysfunction related to insulin resistance initiates early in life and involves impaired vasodilation, loss of arterial distensibility^(57)^, which is in full agreement with the present findings. In line with our findings, a cross-sectional study in 1327 healthy subjects indicated that physical activity combined with a healthy diet characterised by high consumption of fruits, vegetables, olive oil and nuts may produce an improvement in AIx^(58,59)^. Moreover, another study found that daily vegetable consumption is associated with improved AIx in a population at increased risk for CVD^(60)^. A randomised control trial also demonstrated that increased fruit and vegetable consumption improved AIx in men with established CVD^(61)^. Fish and seafood are rich in *n*-3 PUFAs, which may reduce cardiovascular risk via favourable effects exerted on arterial structure and function. A longitudinal study in eighty-six patients with rheumatoid arthritis investigated the association between *n*-3 PUFAs from fish consumption and AIx^(62)^. It was shown that subjects with overall higher fish intake and use of fish oil supplement presented with a lower AIx, which is in line with our finding. Other studies investigated the relationship between AIx and *n*-3 fatty acids with supplements. Results showed no association between AIx and *n*-3 supplements amongst both young and older healthy subjects^(63)^ and healthy Japanese men^(64)^.

The gold-standard biomarker for arterial stiffness assessment in clinical practice is PWV. Only a few studies have investigated the associations between DPs and PWV. The prospective cohort SU.VI.MAX study conducted in 1026 middle-aged subjects with 7·5 years of follow-up investigated the relationship between PWV and dietary patterns. There was a significantly positive association between PWV and a DP characterised by high consumption of meat, poultry, processed meat and alcohol and low fibre intake. No other statistical significant association was observed between any dietary pattern and PWV^(21)^. On the other hand, a much smaller cross-sectional study in seventy middle-aged subjects indicated that a DP characterised by high consumption of fruits, vegetables, pulses, seafood and seaweed, is inversely associated with arterial stiffness measured by PWV^(22)^. However, in the present study, no association was observed between any of the DPs and PWV. **DP 5** includes increased consumption of heavy alcoholic drinks and non-diet soft drinks with sugar. In the present study no significant association between increased consumption of heavy alcoholic drinks and non-diet soft drinks with sugar with any marker of vascular structure and function was observed, which is in contrast with the above-mentioned previous findings. However, this may be due to very low consumption of these food items in our population which might have affected the present findings. Previous studies have shown a relationship between alcoholic drinks, non-diet soft drinks with sugar and vascular markers. For example, cross-sectional studies have investigated the possible association between alcohol consumption and arterial stiffness amongst men and women^(65–68)^. Results are not solid as there are studies showing a J-shaped or U-shaped association^(65,67,68)^, whereas others showing a direct increase in arterial stiffness development as alcohol consumption increases^(66)^. Also, a Japanese longitudinal study observed an unfavourable effect of alcohol on PWV^(69)^. Furthermore, in a meta-analysis showed a significant association between consumption of non-diet soft drinks with sugar and BP and, also, it was shown that individuals with the highest intake (≥1 glass/d) had a 12 % higher risk of hypertension compared with those who had the lowest intake (none)^(70)^.

The current study has both strengths and limitations. The main strength is related to the large sample size consisting of individuals at high CVD risk having thorough assessment of a remarkable number of microcirculation vascular markers reflecting different types of the arterial disease. Furthermore, the assessment of several health and lifestyle parameters allowed adjusting for several confounders. One important limitation is that the study has a cross-sectional design and therefore is not suitable for establishing a cause–effect relationship. In addition, it is known that DPs are quite different regarding their nutrient content and it is possible that DP2 and DP3 characterised by high intake of processed meat and cheese, are higher in their sodium content compared to other derived DPs, leading to their positive association with blood pressure indices. Another important limitation concerns the known methodological constraints of PCA in order to extract *a posteriori* DPs, as it used many subjective decisions. Lastly, the use of 24 h-dietary recalls based on participant’s memory are prone to recall bias and socially desirable answers, but most importantly to risk of underreporting.

## Conclusions

In a group of individuals at CVD risk, adherence to healthy DPs (characterised by increased consumption of whole grains, white meat, vegetables, fruits, fish and seafood and low consumption of sugar) was inversely associated with common carotid compliance, distensibility and arterial stiffness, whereas other DPs (characterised by increased consumption of refined grains, red meat, processed meat, full-fat cheese and butter and low consumption of low-fat dairy product) was positively associated with BP. Future studies are needed to confirm the results of the current study and to determine the effect of dietary habits and food preferences in relation to several pathophysiological conditions of vascular dysfunction.

## Supplementary material

For supplementary material accompanying this paper visit https://doi.org/10.1017/S1368980021003499. click here to view supplementary material
