# Supplementary material for: Associations of dietary patterns with blood pressure and markers of subclinical arterial damage in adults with risk factors for CVD
Source: Public Health Nutr. 2021 Aug 16;24(18):6075–84. doi: 10.1017/S1368980021003499 (PMC11148598; doi:10.1017/S1368980021003499)
Supplement: Supplementary file 1 [file S1368980021003499sup001.docx]

**STROBE Checklist**

|  | Item No | Recommendation |
| --- | --- | --- |
| **Title and abstract** | 1 | (*a*) Indicate the study’s design with a commonly used term in the title or the abstract  ***Title page, abstract (page 1)*** |
|  |  | (*b*) Provide in the abstract an informative and balanced summary of what was done and what was found  ***Abstract, page 1*** |
| Introduction | | |
| Background/rationale | 2 | Explain the scientific background and rationale for the investigation being reported  ***Introduction, page 2-3*** |
| Objectives | 3 | State specific objectives, including any prespecified hypotheses  ***Introduction, page 3*** |
| Methods | | |
| Study design | 4 | Present key elements of study design early in the paper  ***Study design and population, page 3-4*** |
| Setting | 5 | Describe the setting, locations, and relevant dates, including periods of recruitment, exposure, follow-up, and data collection  ***Study design and population, page 3-4*** |
| Participants | 6 | (*a*) Give the eligibility criteria, and the sources and methods of selection of participants. Describe methods of follow-up  ***Study design and population, page 3-4*** |
|  |  | (*b*) For matched studies, give matching criteria and number of exposed and unexposed  ***Not applicable*** |
| Variables | 7 | Clearly define all outcomes, exposures, predictors, potential confounders, and effect modifiers. Give diagnostic criteria, if applicable  ***Materials and methods, pages 4-5*** |
| Data sources/ measurement | 8* | For each variable of interest, give sources of data and details of methods of assessment (measurement). Describe comparability of assessment methods if there is more than one group  ***Definition of CVD risk factors, Blood pressure measurement, Estimation of Arterial Stiffness, Compliance and Distensibility, Estimation of Pressure Wave Reflections, Dietary Assessment, pages 4-5*** |
| Bias | 9 | Describe any efforts to address potential sources of bias |
| Study size | 10 | Explain how the study size was arrived at  ***No sample size calculation was performed; this is a cohort study that used a convenience sample in terms of size*** |
| Quantitative variables | 11 | Explain how quantitative variables were handled in the analyses. If applicable, describe which groupings were chosen and why  ***Statistical analysis, pages 5-6*** |
| Statistical methods | 12 | (*a*) Describe all statistical methods, including those used to control for confounding  ***Statistical analysis, pages 5-6*** |
|  |  | (*b*) Describe any methods used to examine subgroups and interactions  ***Statistical analysis, pages 5-6*** |
|  |  | (*c*) Explain how missing data were addressed  ***Not applicable*** |
|  |  | (*d*) If applicable, explain how loss to follow-up was addressed  ***Not applicable*** |
|  |  | (*e*) Describe any sensitivity analyses  ***Statistical analysis, page 5-6*** |
| Results | | |
| Participants | 13* | (a) Report numbers of individuals at each stage of study—eg numbers potentially eligible, examined for eligibility, confirmed eligible, included in the study, completing follow-up, and analysed  ***Results, page 6*** |
|  |  | (b) Give reasons for non-participation at each stage  ***Not applicable*** |
|  |  | (c) Consider use of a flow diagram  ***Not applicable*** |
| Descriptive data | 14* | (a) Give characteristics of study participants (eg demographic, clinical, social) and information on exposures and potential confounders  ***Results, page 6*** |
|  |  | (b) Indicate number of participants with missing data for each variable of interest  ***Results, page 6*** |
|  |  | (c) Summarise follow-up time (eg, average and total amount)  ***Not applicable*** |
| Outcome data | 15* | Report numbers of outcome events or summary measures over time  ***Results, page 7*** |
| Main results | 16 | (*a*) Give unadjusted estimates and, if applicable, confounder-adjusted estimates and their precision (eg, 95% confidence interval). Make clear which confounders were adjusted for and why they were included  ***Results, page 6-7*** |
|  |  | (*b*) Report category boundaries when continuous variables were categorized  ***Results, page 6-7*** |
|  |  | (*c*) If relevant, consider translating estimates of relative risk into absolute risk for a meaningful time period  ***Not applicable*** |
| Other analyses | 17 | Report other analyses done—eg analyses of subgroups and interactions, and sensitivity analyses  ***Not applicable*** |
| Discussion | | |
| Key results | 18 | Summarise key results with reference to study objectives  ***Discussion, page 7*** |
| Limitations | 19 | Discuss limitations of the study, taking into account sources of potential bias or imprecision. Discuss both direction and magnitude of any potential bias  ***Discussion, page 10*** |
| Interpretation | 20 | Give a cautious overall interpretation of results considering objectives, limitations, multiplicity of analyses, results from similar studies, and other relevant evidence  ***Discussion, pages 7-10*** |
| Generalisability | 21 | Discuss the generalisability (external validity) of the study results  ***4. Discussion, pages 7-10*** |
| Other information | | |
| Funding | 22 | Give the source of funding and the role of the funders for the present study and, if applicable, for the original study on which the present article is based  ***Source of funding, Title page, page 1*** |
